# Supplementary material for: Coupled reaction equilibria enable the light-driven formation of metal-functionalized molecular vanadium oxides
Source: Nat Commun. 2023 Sep 9;14:5563. doi: 10.1038/s41467-023-41257-y (PMC10492840; doi:10.1038/s41467-023-41257-y)
Supplement: Supplementary file 1 — Supplementary Information [file 41467_2023_41257_MOESM1_ESM.pdf]

Supplementary Information

Table of Contents

1. Instrumentation 1
2. Experimental Section and Characterization 4
   2.1. Synthesis of Compound 1: $(nBu_4N)_4[(MgCl)_2V^{IV}V^{V}_{11}O_{32}Cl] \cdot CH_3CN$ (= $(nBu_4N)_4\{Mg_2V_{12}\}$): 4
   2.2. UV/Vis-NIR spectroscopy 4
   2.3. EPR spectroscopy 5
   2.4. ^1H NMR spectroscopy 5
   2.5. ^{51}V NMR spectroscopy 6
   2.6. ATR-IR-spectroscopy 7
   2.7. Thermogravimetric analysis 7
   2.8. High-resolution ESI mass spectrometry 8
   2.9. Crystallographic Details 9
3. Mechanistic studies 11
   3.1. NMR spectroscopy 11
   3.2. UV-Vis-NIR spectroscopy 14
   3.3. Electrochemistry 16
   3.4. Mg^{2+} replacement with Ca^{2+} 16
4. Theoretical calculations 18
5. References 19

1. Instrumentation

Single-crystal X-Ray diffraction (scXRD) was performed on a Bruker D8 Quest single-crystal diffractometer with a PHOTON II detector using Mo-Kα radiation (wavelength λ = 0.71073 Å).

Attenuated total reflectance-Fourier-transform infrared spectroscopy (ATR-FT-IR) was performed on a Bruker Alpha II equipped with an ATR Platinum Diamond unit. The data were recorded with 24 scans at a resolution of 4 cm^{-1}.

UV/Vis-NIR spectroscopy was performed on a V-670 JASCO UV-VIS-NIR Spectrometer. Quartz glass cuvettes (d = 10.0 mm) were used for all measurements.

NMR spectroscopy: NMR spectra were recorded on a Bruker AVANCE DRX 400 MHz spectrometer (Bruker Biospin GmbH, Rheinstetten, Germany) equipped with 5 mm inverse probe head operating at 400.13 MHz ^1H frequency and 105.51 MHz ^{51}V frequency at ambient temperature. Stimulated echo sequence with bipolar gradient pulses and a longitudinal eddy current delay was used for the ^1H diffusion ordered spectroscopy (DOSY) experiments. The gradient strength was linearly incremented in 16 steps from 2 % to 95 % of the maximum gradient strength. The diffusion time and the gradient pulse length for all samples were 100 ms and 2.8 ms with 2 s recycle delay, respectively. After Fourier transformation and baseline

correction, the diffusion dimension of the 2D DOSY spectra was processed using the Topspin 1.3 software package (2007, patchlevel 8, Bruker Biospin GmbH, Rheinstetten, Germany). The diffusion analysis was performed using the Topspin T1/T2 relaxation package.

The ^{51}V Total COrrelation SpectroscopY (TOCSY) experiment was performed using the *mlevph* pulse sequence with a MLEV-17 spin-lock pulse of 18 ms duration recording 512 data points in the direct dimension with 512 increments, 32 transients and a recycle delay of 0.1 s. Sweep widths of 50 ppm and an offset of -574 ppm were used in both dimensions. Zero filling to 1024 data points and broadening of 60 Hz was applied before Fourier transformation. The ^{1}H chemical shift values (δ) are given in part per million (ppm) using residual solvent protons (δH = 1.94 ppm for CD_3CN, δH = 2.50 ppm for $(CD_3)_2SO$). ^{51}V NMR spectra were referenced to external $VOCl_3$ + 5% C_6D_6 at 0 ppm.

High resolution electrospray ionization mass spectrometry (ESI MS) was carried out in on an Agilent 6545 QTOF-HRAM-MS system in negative ion mode at a drying gas temperature of T = 180 °C.

Thermogravimetric analysis (TGA) was carried out on a NETZSCH TG 209F1 analyser at a heating rate of 5.0 K min^{-1} in a range between 30 and 700 °C under air in an Al_2O_3 crucible.

Electron paramagnetic resonance (EPR) spectroscopy was performed on a X-band Bruker Magnettech ESR5000 spectrometer at room temperature on a microcrystalline sample of **{Mg_2V_{12}}**. The solid sample was filled in a glass EPR-tube (diameter 3 mm), the measurement range was 225 to 450 mT with a modulation of 1mT, a microwave power of 25 mW and a sweep time of 120s. The SpinCount software option was used to calculate the spin quantity in the sample.

Irradiation setup: a 20 W broadband LED light source from REV Ritter GmbH, model number: ESFL-6620-ST was used. The following parameters are provided by the manufacturer: nominal power: 20 W; number of LEDs: 28; luminous flux 1700 lm; luminous efficacy 85 lm/W. The following parameters were measured by a cosine corrector: 2D radiometry corrected power: 4.98 W; optical power: 4.97 W; photon flux: 0.223 mmol/s. For wavelength-selective irradiations, 405 nm LEDs (Edison, 3.2 V, 500 mA) or 470 nm LEDs (Quadrios, 3.0 V, 20 mA) were used.

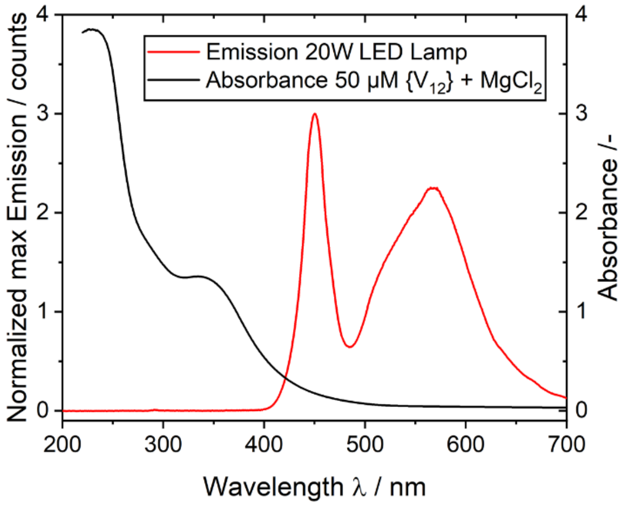

Supplementary Figure 1: Normalized emission spectrum of the 20 W LED light source, and comparison with the absorbance of the standard {V_{12}} / $MgCl_2$ / MeCN reaction solution.

Solution electrochemistry: DC cyclic voltammetry (CV) experiments were performed on a Pine Research WaveDriver 200 electrochemical workstation equipped with a standard three-

electrode arrangement: working electrode: glassy carbon electrode (d = 3.0 mm), quasi reference electrode: Ag wire (in a glass frit containing electrolyte solution), counter electrode: Pt wire. All potentials are quoted relative to the ferrocene/ferrocenium internal standard. All experiments were performed in anhydrous MeCN using $n\mathrm{Bu_4NPF_6}$ (0.1 M) as supporting electrolyte. The solutions were purged with desired gas (Ar or O_2) for at least 25 min and kept under a slight positive Ar or O_2 pressure while performing the experiments.

Bond Valence Sum Calculation was performed using the program Visualization for Electronic and Structural Analysis (VESTA),[1] as outlined in reference [2] using the published parameters from the file bvparm2016.cif (https://www.iucr.org/resources/data/datasets/bond-valence-parameters)

Theoretical calculations: Geometry optimizations of the clusters were carried out using density functional theory calculations with the B3LYP[3,4] functional combined with the def2-SVP basis set.[5] Solvation effects were considered using the SMD implicit solvation model (acetonitrile).[6] UV/Vis absorption spectra were computed at all stable local minima using the B3LYP functional and def2-SVP basis set. Implicit solvation was implemented using C-PCM (acetonitrile).[7] .All calculations were performed using the Gaussian 16 package.[8]

2. Experimental Section and Characterization

All chemicals were purchased from Sigma Aldrich, VWR or Alfa Aesar and were of reagent grade. The chemicals were used without further purification unless stated otherwise. All experiments were performed under inert conditions in the glovebox. All solvents in the glovebox were oxygen and water free. (nBu$_4$N)$_3$(NMe$_2$H$_2$)$_2$[V$_{12}$O$_{32}$Cl)] x CH$_3$CN (= **{V$_{12}$}**) was synthesized as described in reference.[9]

2.1. Synthesis of Compound 1: (nBu$_4$N)$_4$[(MgCl)$_2$V^{IV}V$^{V}_{11}$O$_{32}$Cl] · CH$_3$CN (= (nBu$_4$N)$_4${Mg$_2$V$_{12}$})

The synthesis of **1** was performed in a glovebox unfder argon atmosphere: in a 25 mL round-bottom flask 0.200 g (0.100 mmol) (nBu$_4$N)$_3$(NMe$_2$H$_2$)$_2$[V$_{12}$O$_{32}$Cl)] x CH$_3$CN, 0.056 g (0.200 mmol) nBu$_4$NCl and 0.0400 g (0.420 mmol) anhydrous MgCl$_2$ were dissolved in 12 mL water-free, deaerated acetonitrile and stirred at room temperature. After four hours of stirring, the yellow solution was filtered through a glass Pasteur pipette filled with glass wool. Diffusion crystallisation with diethyl ether was setup and the samples were exposed to light, either via the glovebox fluorescent lamp or the broadband visible light source described in Supplementary Figure 1. After two days, dark green crystals of **1** were obtained, filtered, washed twice with acetone and diethyl ether and dried under vacuum. Molecular weight: 2289.15 g/mol, yield: 0.146 g (0.064 mmol, 63.8% based on V).

2.2. UV/Vis-NIR spectroscopy

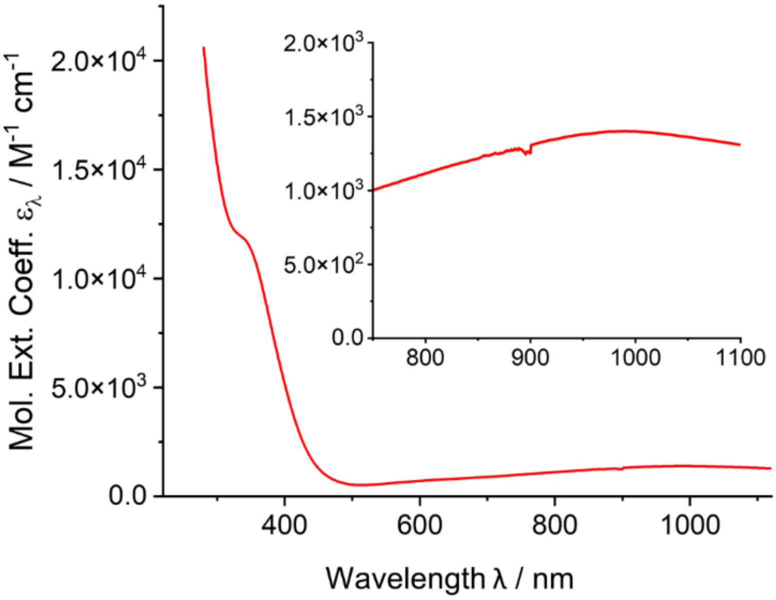

Supplementary Figure 2: UV-Vis-NIR spectrum of a DMF solution of **1** (56 µM)**:** ε_{338}: 11,875 M^{-1} cm^{-1}; ε_{991}: 1,400 M^{-1} cm^{-1}. Inset: magnified view of the region between 750 nm and 1100 nm, showing the characteristic IVCT transition indicative of the presence of a mixed-valent V$^{IV/V}$ species.

2.3. EPR spectroscopy

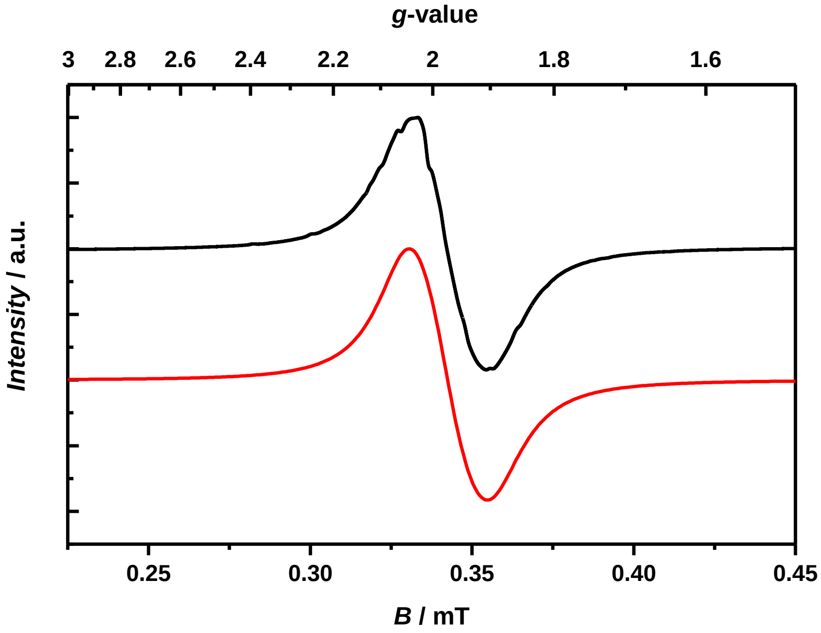

Supplementary Figure 3: Black: Solid state CW-EPR spectrum of **1** between 225 mT to 450 mT with a modulation of 1mT, a microwave power of 25 mW and a sweep time of 120 s. Red: simulation with S= ½, g_1 = 1.904(1), g_2 = 1.979(1), g_3 = 2.049(1) and isotropic line width = 0.696(5) GHz.

2.4. ^{1}H NMR spectroscopy

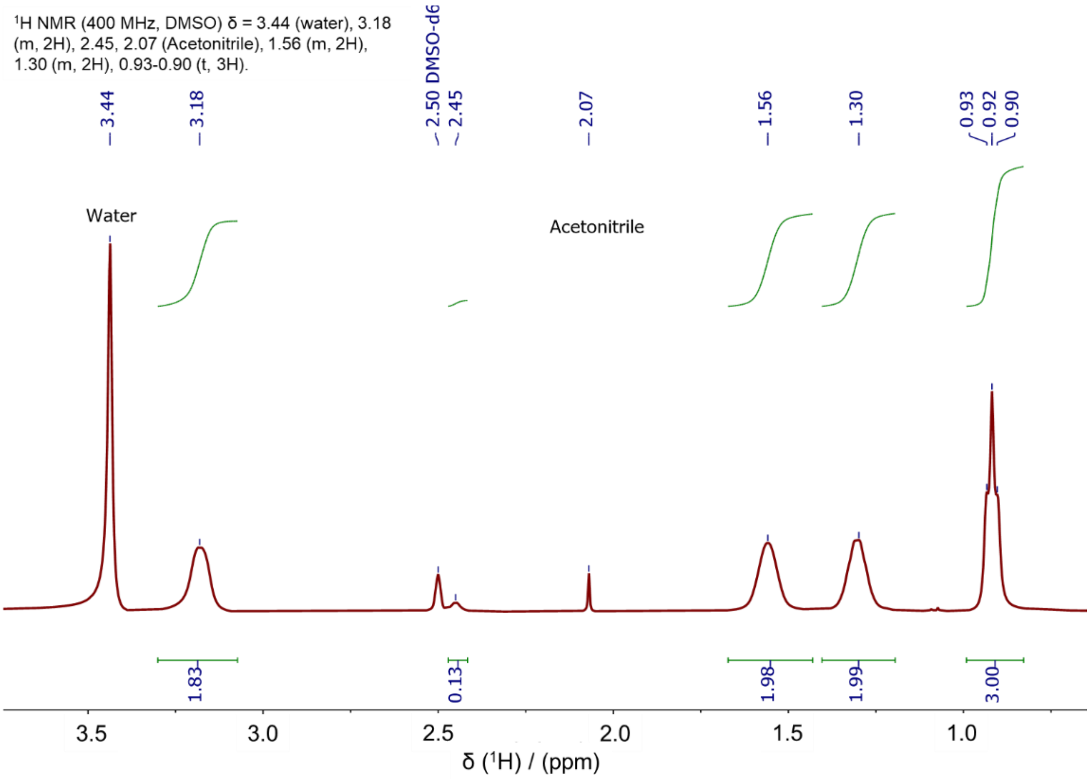

Supplementary Figure 4: ^{1}H NMR spectrum of **1**. Conditions: solvent: DMSO-d_6, 400 MHz, 64 scans. Signal assignments: δ (ppm) **=** 3.44 (s, H_2O); 3.18 (m, 2 H, nBu_4N^+); 2.45 (impurity); 2.07 (s, acetonitrile); 1.56 (m, 2 H, nBu_4N^+); 1.30 (s, 2 H, nBu_4N^+); 0.92 (t, 3 H, nBu_4N^+)

2.5. ^{51}V NMR spectroscopy

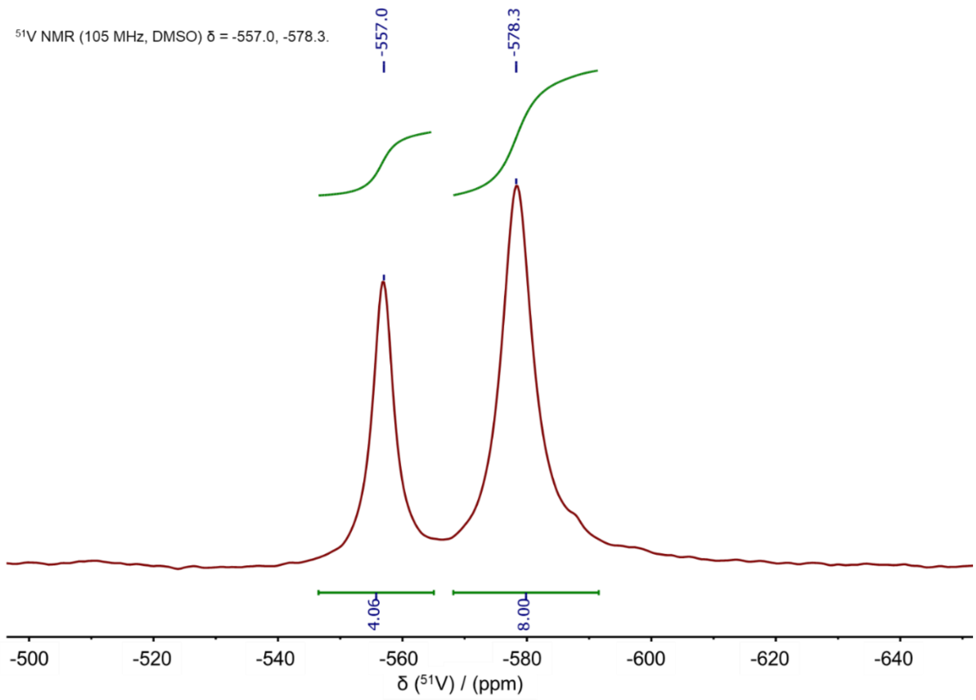

Supplementary Figure 5: ^{51}V NMR spectrum of **1.** Conditions: [**1**] ca. 7.5 mM in DMSO-d_6,10,000 scans; 105 MHz, δ (ppm) = - 557.0 (s, 4V); - 578.3 (s, 8V).

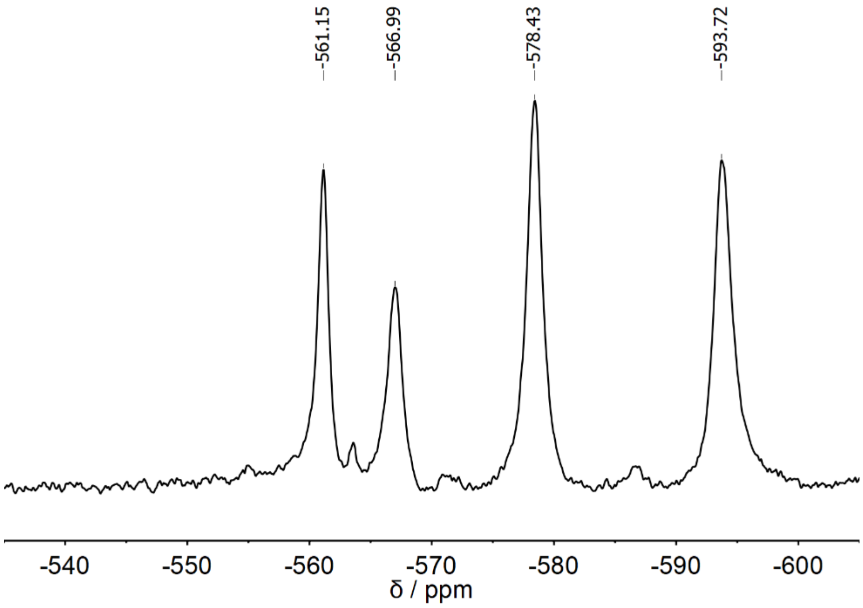

Supplementary Figure 6: ^{51}V NMR spectrum of **1**, {Mg_2V_{12}} in MeCN, indicating that upon dissolving {Mg_2V_{12}} in MeCN, one Mg^{2+} is released and {MgV_{12}} is re-formed. Conditions: [**1**] ca. 5.0 mM in MeCN.

2.6. ATR-IR-spectroscopy

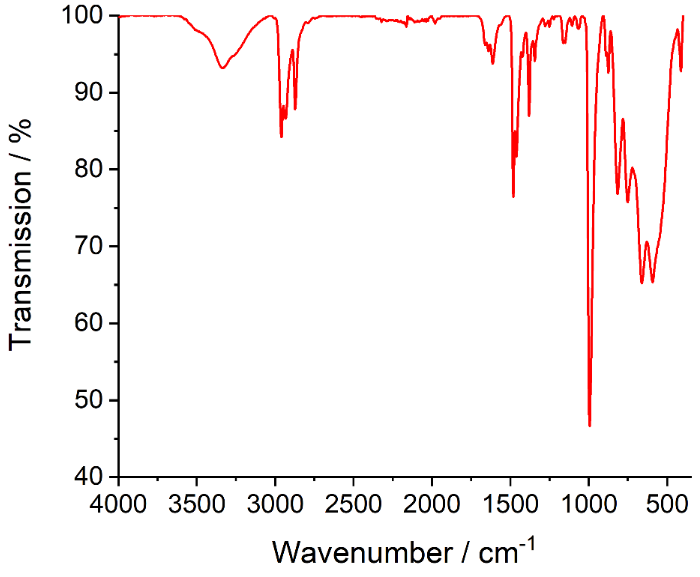

Supplementary Figure 7: ATR-FT-IR spectrum of compound **1**. Characteristic IR bands (in cm^{-1}): 3336; 2961 (C-H stretching, alkane); 2934 (C-H stretching, alkane); 2874 (C-H stretching, alkane); 1659; 1641; 1613; 1482; 1461; 1381;1422; 1345; 1278; 1251; 1163; 1151; 1107; 1067; 995 (symmetric V=O, terminals Oxygen); 890; 875 (anti-symmetric stretching V-O); 817; 752 (V-O-V, symmetric); 661 (V_3-$O_{\mu 3}$, asymmetric); 593; 412.

2.7. Thermogravimetric analysis

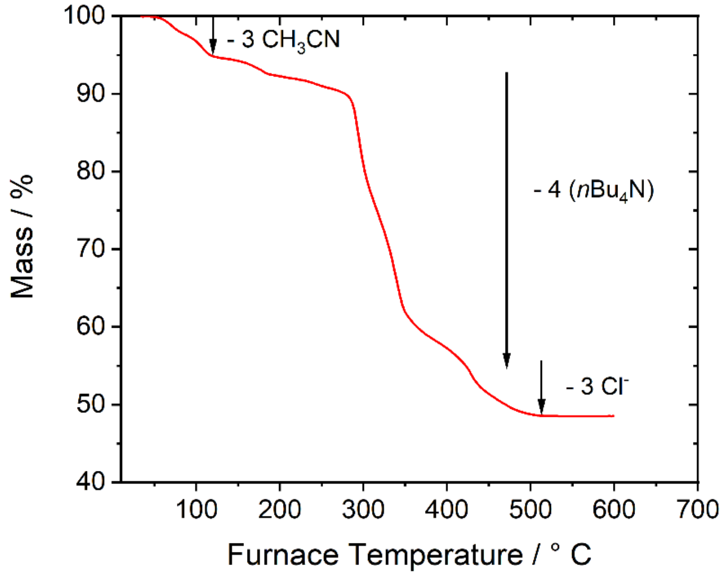

Supplementary Figure 8: Thermogravimetric analysis (under air) of compound **1**. A weight loss of 5.01 wt.-% between 30 °C and 116 °C is shown, which corresponds to the loss of three acetonitrile (calc.: 5.21 wt.-%). A further weight loss of 41.97 wt.-% between 116 °C and 434 °C, corresponds to the loss of four tetra-n-butylammonium cations (calc.: 41.04 wt.-%). The loss of 4.47 wt.% above 436 °C can be assigned to the loss of the three chlorides (calc.: 4.50 %).

2.8. High-resolution ESI mass spectrometry

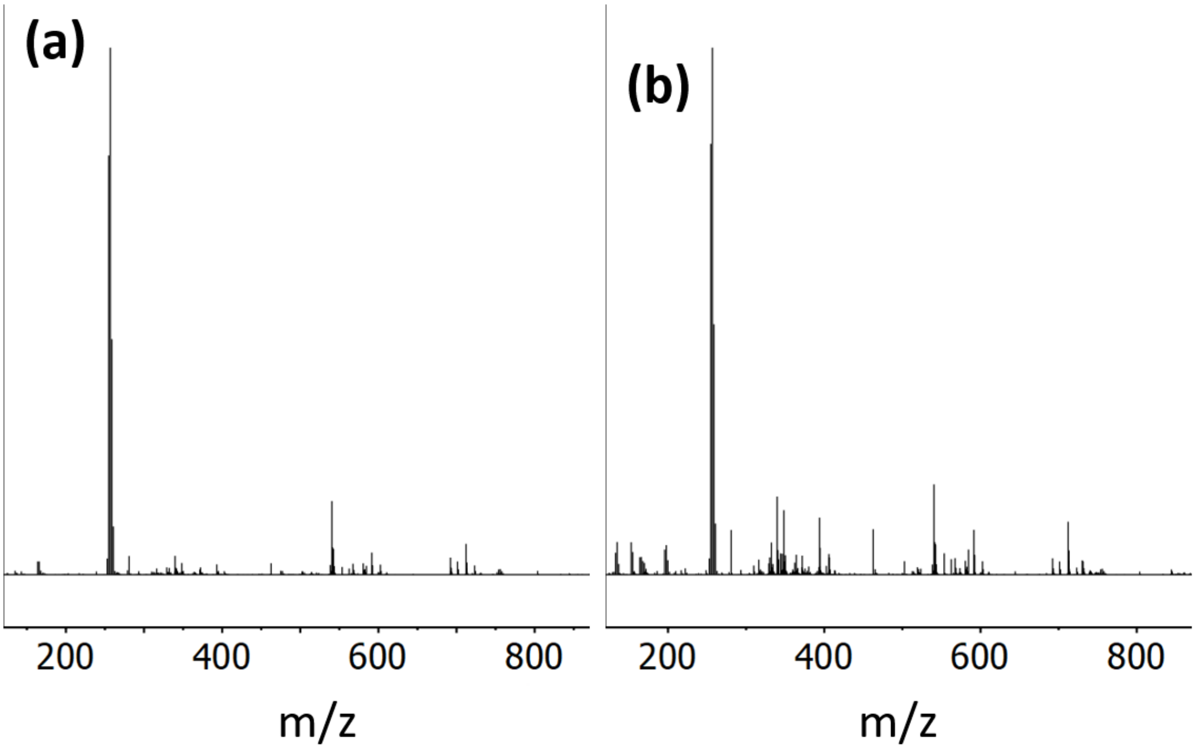

Supplementary Figure 9: high-resolution negative-ion mode ESI mass spectra of (a) the reference sample, $\{V_{12}\}$ (0.05 mM) dissolved in MeCN; (b) the reaction solution containing $\{V_{12}\}$ (0.05 mM) and $MgCl_2$ (0.21 mM) dissolved in MeCN. Detailed peak assignment, see below.

Supplementary Table 1: ESI MS peak assignments for $\{V_{12}\}$ and the reaction mixture, $\{V_{12}\}+MgCl_2$

**ESI-MS of reaction mixture ($\{V_{12}\}$ + $MgCl_2$ in MeCN)**		
**observed m/z**	**calculated m/z**	**peak assignment**
280.785	280.801	$[V_6O_{16}]^{2-}$
339.746	339.754	$[V_7O_{18}Cl]^{2-}$
371.727	371.735	$[V_8O_{21}]^{2-}$
394.073	394.052	$\mathbf{[MgV_{12}O_{32}Cl]^{3-}}$
462.653	462.670	$[V_5O_{13}]^{-}$
591.590	591.582	$\mathbf{[HMgV_{12}O_{32}Cl]^{2-}}$
701.208	701.236	$(^{n}Bu_4N)[V_{12}O_{32}Cl]^{2-}$
**ESI-MS of reference sample ($\{V_{12}\}$ in MeCN)**		
280.785	280.801	$[V_6O_{16}]^{2-}$
339.746	339.754	$[V_7O_{18}Cl]^{2-}$
371.727	371.735	$[V_8O_{21}]^{2-}$
462.653	462.670	$[V_5O_{13}]^{-}$
580.563	580.597	$\mathbf{H_3[V_{12}O_{32}Cl]^{2-}}$
701.208	701.236	$\mathbf{(^{n}Bu_4N)[V_{12}O_{32}Cl]^{2-}}$

Note that the vanadate fragments observed are most likely artefacts due to the ionization / gas phase transfer process and have been observed for the native $\{V_{12}\}$[9] as well as other related POM species previously.[10]

2.9. Crystallographic Details

Suitable single crystals were mounted onto a microloop using Fomblin oil. X-ray diffraction intensity data were measured at 150 K on a Bruker D8 QUEST diffractometer λ(MoKα = 0.71073 Å) equipped with a graphite monochromator. Structure solution was carried out using SHELX-2013[11] package through OLEX2.[12] Corrections for incident and diffracted beam absorption effects were applied using empirical methods.[13] Structures were solved by a combination of direct methods and difference Fourier syntheses and refined against F2 by the full matrix least-squares technique. Non-hydrogen atoms were refined anisotropically. Hydrogen atoms were added to carbon atoms using a riding model. The metal oxo framework was refined fully anisotropically. The $n\mathrm{Bu_4N^+}$ counter cations were severely disordered, and restraints (SIMU and DELU) were applied. CCDC 2240239 contains the supplementary crystallographic data for this paper. These data can be obtained free of charge from The Cambridge Crystallographic Data Centre via www.ccdc.cam.ac.uk/structures.

Supplementary Table 2: Crystallographic Parameters for **1**

**CCDC Number**	2240239
Empirical formula	$(nBu_4N)_4[Mg_2Cl_3V_{12}O_{32}] \cdot CH_3CN$
Formula weight	2289.15
Temperature/K	150.0
Crystal system	monoclinic
Space group	$P2_1/c$
a/Å	24.3414(9)
b/Å	16.7474(7)
c/Å	24.4623(9)
α/°	90
β/°	94.6107(17)
γ/°	90
Volume/Å^3	9939.9(7)
Z	4
ρ_{calc}g/cm^3	1.530
μ/mm^{-1}	1.238
F(000)	4740.0
Radiation	MoKα (λ = 0.71073)
2Θ range for data collection/°	3.616 to 54.374
Index ranges	-31 ≤ h ≤ 31, -21 ≤ k ≤ 21, -31 ≤ l ≤ 31
Reflections collected	332994
Independent reflections	22031 [R_{int} = 0.0771, R_{sigma} = 0.0266]
Data/restraints/parameters	22031/1591/1098
Goodness-of-fit on F^2	1.104
Final R indexes [I>=2σ (I)]	R_1 = 0.0518, wR_2 = 0.1345
Final R indexes [all data]	R_1 = 0.0650, wR_2 = 0.1449
Largest diff. peak/hole / e Å^{-3}	1.58/-0.88

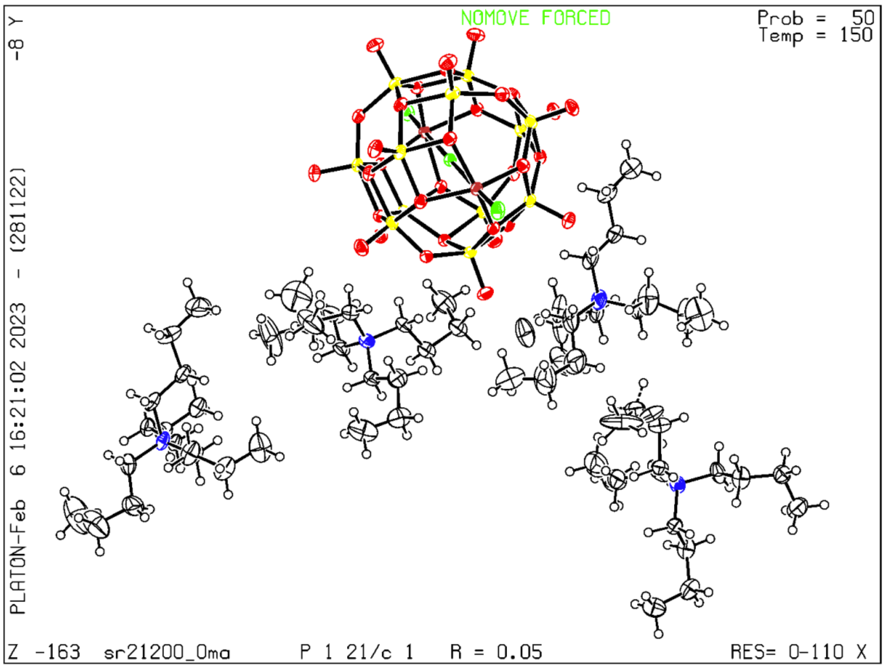

Supplementary Figure 10: ORTEP illustration of the single-crystal XRD structure of $\{Mg_2V_{12}\}$. Probability ellipsoids drawn at 50 % probability.

Bond Valence Sum (BVS) Calculations

BVS calculations were carried out on the single-crystal XRD structure of 1 using the following equations.

$$S_{ij} = \exp\left(\frac{R_0 - R_{ij}}{B}\right) \quad \text{Eq. S1}$$

$$V_i = \sum_j S_{ij} \quad \text{Eq. S2}$$

$$G_{ii} = \sqrt{\frac{1}{N}\sum_{i=1}^{N}\left(V_i - V_i^0\right)^2} \quad \text{Eq. S3}$$

S_{ij} = bond valence; R_0 = bond valence constant parameter; B = bond valence constant parameter; i = atom; j = neighboring atoms; V_i = formal oxidation state of the atom i; G_{ii} = global instability index; N = Number of atoms; V_i^0 = Oxidation state of the formal ionic charge.

R_0 (V(V) to O(-II)) = 1.803 Å with B = 0.37 were used. V_i^0 = 5 was used as formal ionic charge.

The data show that the single reduced V(IV) centre is not localized on a specific vanadium ion at T = 150 K (*i.e.*, the collection temperature for the single-crystal XRD data which is the basis for the BVS analysis).

Supplementary Table 3: Bond Valence Sum calculation summary for the vanadium atoms of **1**.

Vanadium	V_i	G_{ii}	Vanadium	V_i	G_{ii}
V1	5.041	0.04095	**V7**	4.791	0.2089
V2	5.100	0.1002	**V8**	5.029	0.02889
V3	4.986	0.0141	**V9**	4.885	0.1151
V4	5.060	0.06001	**V10**	5.110	0.1099
V5	5.051	0.05133	**V11**	4.922	0.07843
V6	5.073	0.07322	**V12**	5.061	0.06094

3. Mechanistic studies

3.1. NMR spectroscopy

^{1}H and ^{51}V NMR spectra of a solution of 10.0 mg (5.0 µmol) {V_{12}} in acetonitrile-d_3 (500 µL) were recorded. The same experiments were performed for the identical solution also containing 2.0 mg (21.0 µmol) $MgCl_2$. The solution was investigated after storage in the dark and after irradiation (*vide infra*). All experiments were prepared in a glovebox under argon atmosphere and compared with a {V_{12}} and {Mg_2V_{12}} references (recorded under ambient conditions). The NMR spectra are shown below.

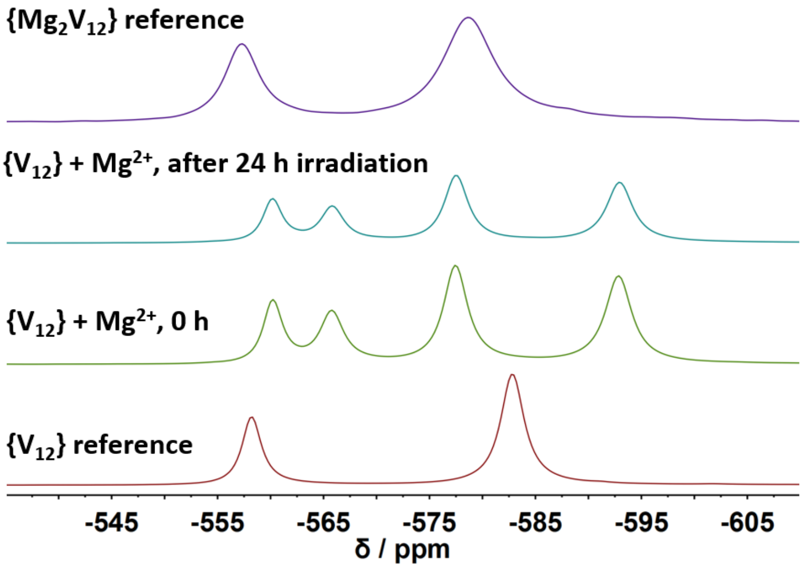

Supplementary Figure 11: ^{51}V NMR spectra of {V_{12}} in acetonitrile upon addition of $MgCl_2$ before and after irradiation for 24 h, in comparison with ^{51}V NMR spectra of the {V_{12}} (in acetonitrile) and {Mg_2V_{12}} (in DMSO) reference compounds. Conditions: [Mg^{2+}] ca. 42 mM, [{V_{12}}] ca. 10 mM.

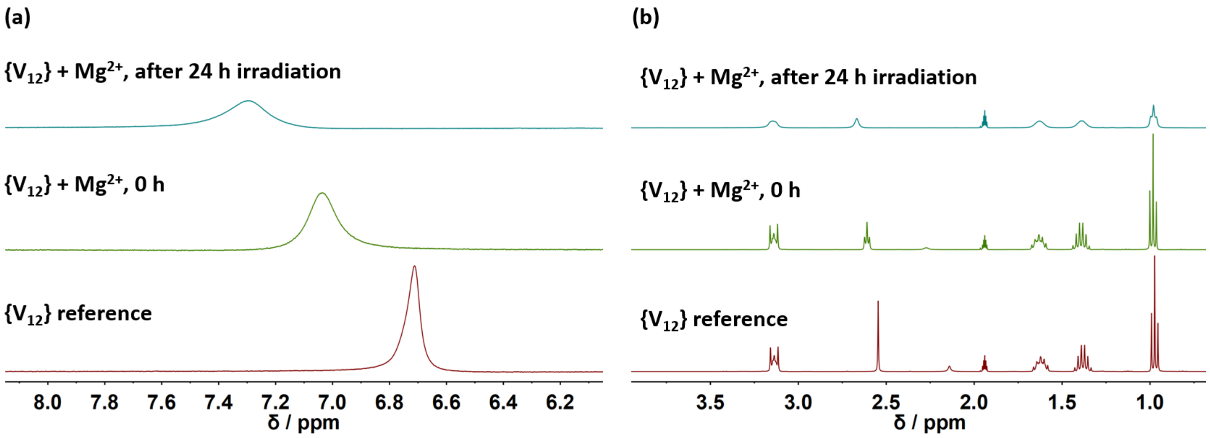

Supplementary Figure 12: ^{1}H NMR spectra ((a) 6 - 8 ppm region, (b) 1 – 3.5 ppm region) of pure {V_{12}} (red) as well as a mixture of {V_{12}} (10 mM) and $MgCl_2$ (42 mM) in CD_3CN immediately after preparation (green), and after 24 h irradiation using a 20 W broadband LED light source (blue). The characteristic low-field shift of the DMA N-H protons upon $MgCl_2$ addition shown in (a) indicate the release of one DMA upon formation of {MgV_{12}} and release of another DMA cation upon light-induced formation of {Mg_2V_{12}}, see main manuscript, Fig. 4 for a schematic illustration of the processes.

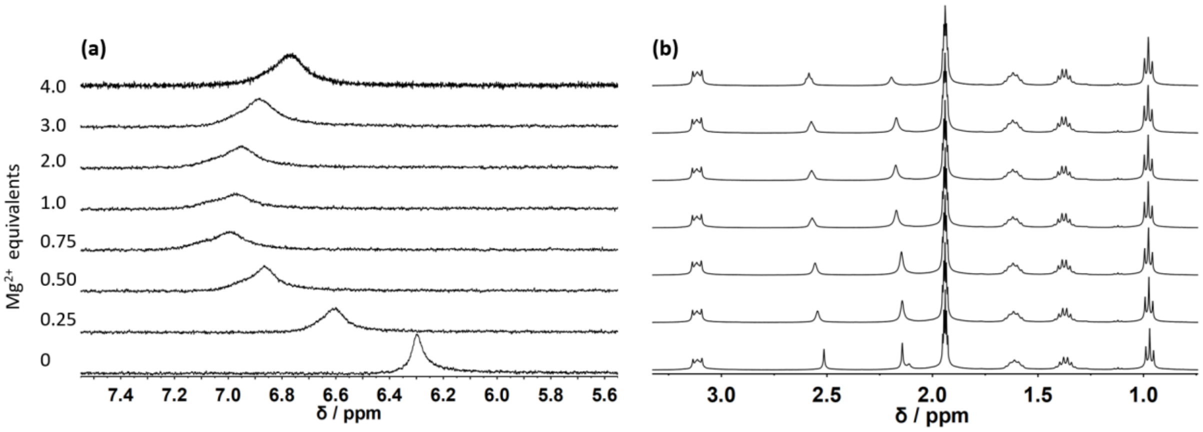

Supplementary Figure 13: stacked ^{1}H NMR spectra ((a) 5.6 – 7.5 ppm region, (b) 0.9 – 3.3 ppm region) of acetonitrile solutions containing $\{V_{12}\}$ and varying Mg^{2+} molar equivalents (between 0 eq. to 4 eq. relative to $\{V_{12}\}$). Upon Mg^{2+} addition, characteristic low-field shifts of the N-H protons of the DMA cations are observed (δ ~ 6.3 ppm to δ ~ 7.0 ppm), indicating a dynamic equilibrium between cluster-bound and "free" DMA. Conditions: $[\{V_{12}\}]$ = 5.0 mM, $[Mg^{2+}]$ = 0 – 21.1 mM, solvent = acetonitrile.

Supplementary Table 4: Summary of the observed ^{1}H and ^{51}V NMR signals.

Sample:	^{1}H NMR chemical shift / ppm	^{51}V NMR chemical shift / ppm
$\{V_{12}\}$ (oxygen-free, CD_3CN)	6.71 (s, 1H), 3.16 – 3.12 (m, 8H), 2.55 (s, 5H), 2.14 (water), 1.66 – 1.58 (m, 8H), 1.43 – 1.34 (m, 8H), 0.99 – 0.96 (t, 12H).	-557.7 (s, 4V), -582.4 (s, 8V).
$\{V_{12}\}$ + $MgCl_2$ (oxygen-free, dark conditions, CD_3CN)	7.04 (s, 2H), 3.16 – 3.12 (m, 8H), 2.62 – 2.60 (t, 5H), 2.27 (s, 1H), 1.67 – 1.59 (m, 8H), 1.44 – 1.35 (m, 8H), 1.00 – 0.96 (t, 12H).	-364.4, -559.8 (s, 2V), -565.3 (s, 2V), -577.0 (s, 4V), -592.3 (s, 4V).
$\{V_{12}\}$ + $MgCl_2$ After visible light irradiation for 24 h (oxygen-free, CD_3CN)	7.29 (s, 2H), 3.15 (m, 8H), 2.67 (s, 5H), 1.63 (m, 8H), 1.40 – 1.38 (m, 8H), 1.00 – 0.96 (t, 12H).	-364.6, -559.8 (s, 2V), -565.4 (s, 2V), -577.1 (s, 4V), -592.4 (s, 4V).
$\{Mg_2V_{12}\}$ reference (ambient, DMSO-d_6)	3.44 (s, H_2O); 3.18 (m, 2 H); 2.45 (n/a); 2.07 (s, acetonitrile); 1.56 (m, 2H); 1.30 (s, 2H); 0.92 (t, 3H)	-557.3, -578.8, -949.8

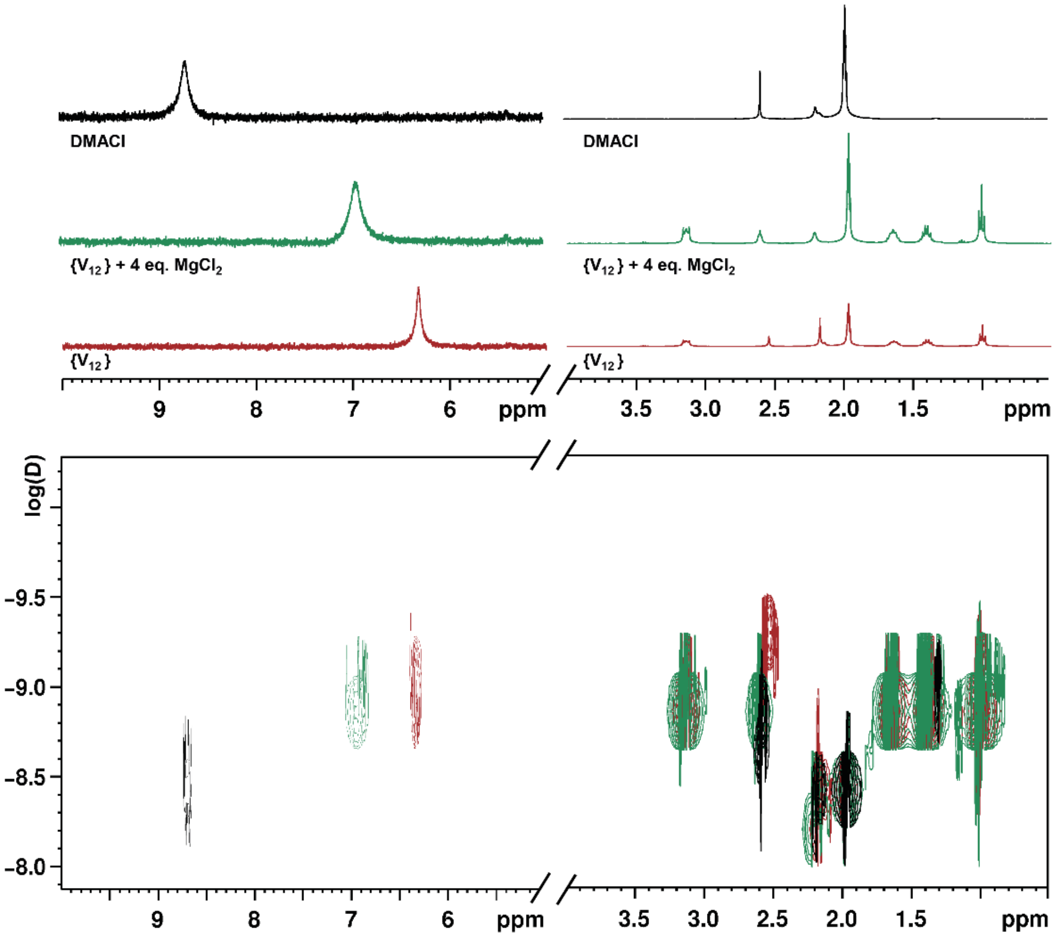

Supplementary Figure 14 Top: ^{1}H NMR spectra of {V_{12}} (red), the {V_{12}} /Mg^{2+} reaction mixture (green) and pure DMA (black) in CD_3CN. The ammonium resonance for the {V_{12}} /Mg^{2+} mixture is located between the resonances of the "free" and the cluster-bound DMA, suggesting a Mg^{2+}-concentration-dependent dynamic equilibrium in solution. The DMA ^{1}H methyl resonance is virtually unaffected in all measurements shown, as the chemical environment for the methyl groups does not significantly change, independent on whether the species is cluster-bound or "free" in solution. **Bottom:** ^{1}H DOSY NMR spectra of {V_{12}} (red), the {V_{12}} /Mg^{2+} reaction mixture (green) and pure DMA (black) overlaid. The data show that the diffusion coefficients decrease in the order "free" DMA < {MgV_{12}} < {V_{12}}. This is expected, as the {MgV_{12}} reaction solution contains a mixture of cluster-bound and "free" DMA (released during Mg^{2+} binding), so that an averaged signal of the two DMA species is observed. Conditions: solvent: CD_3CN, [Mg^{2+}] = 21 mM, [{V_{12}}] = 5.0 mM, [DMA] = 10 mM.

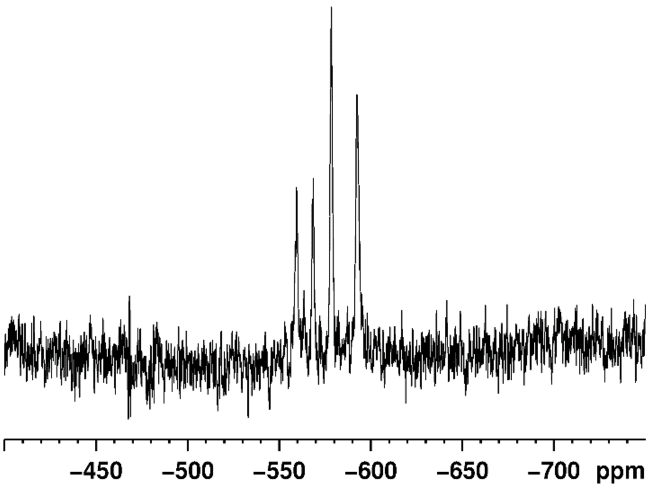

Supplementary Figure 15: [51]V NMR spectrum of {V_{12}} upon addition of $MgCl_2$ at HR ESI MS concentrations. Conditions: solvent: CH_3CN, [Mg^{2+}] = 0.21 mM, [{V_{12}}] = 0.05 mM.

3.2. UV-Vis-NIR spectroscopy

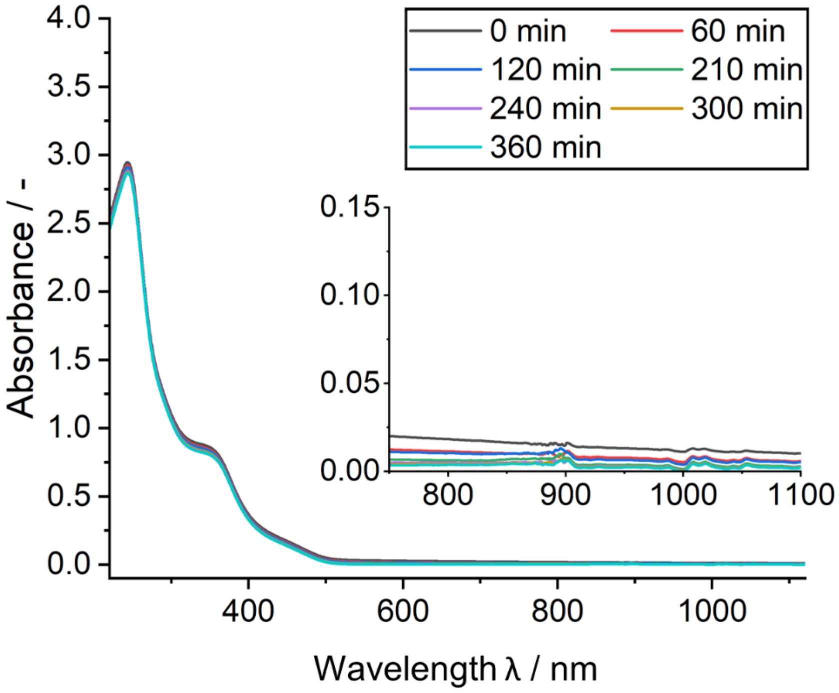

Supplementary Figure 16: Time-lapse UV/Vis spectra of {V_{12}} when irradiated with a broadband high-power LED light source ($P_{nominal}$ = 20 W). Conditions: solvent: acetonitrile, [{V_{12}}] = 50 µM, Ar atmosphere. Note that no reduction (indicated by the characteristic IVCT transitions between 600 nm – 1100 nm) of {V_{12}} is observed.

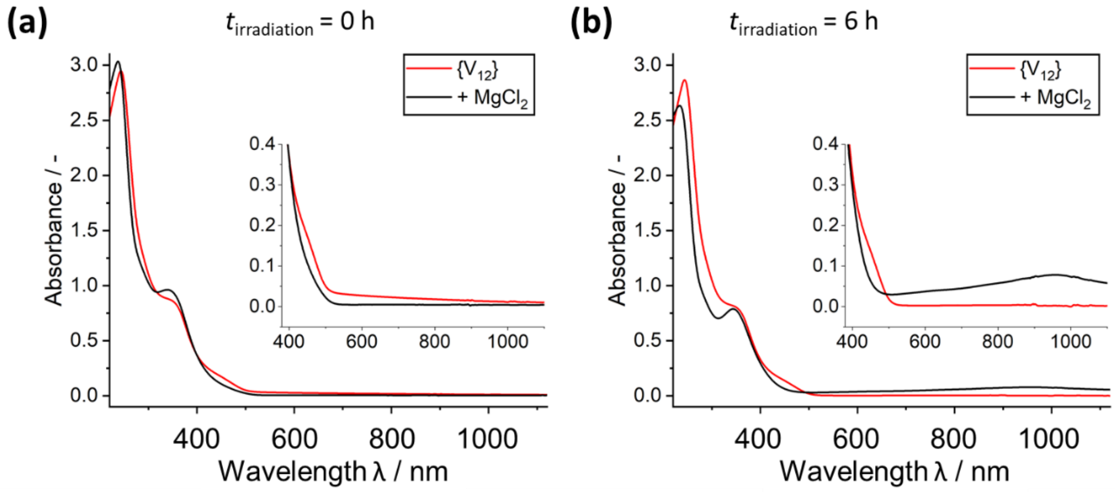

Supplementary Figure 17: UV-Vis-NIR spectra of {V_{12}} in the absence (black) and presence (red) of Mg^{2+}. (a) showing the reaction mixture before irradiation, indicating the in-situ formation of {MgV_{12}} (characteristic changes between 300 nm to 500 nm). (b) after 6 h irradiation, showing the formation of the 1-electron-reduced {Mg2V12} (by the characteristic IVCT transitions between 700 nm – 1100 nm). Conditions: solvent: acetonitrile, [{V_{12}}] = 50 µM, [$MgCl_2$] = 211 µM, Ar atmosphere.

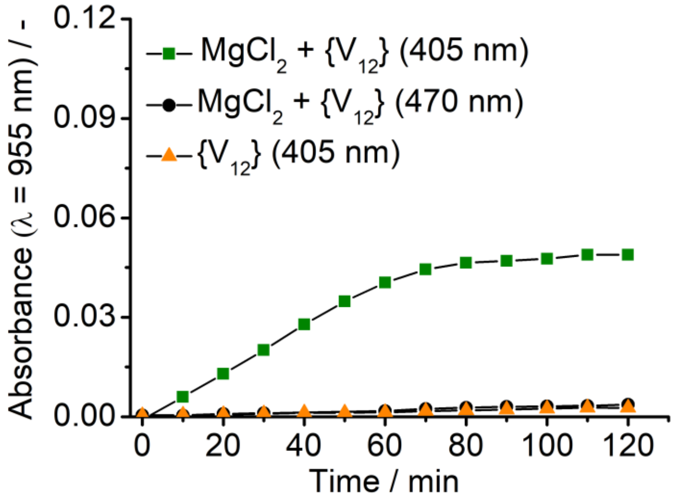

Supplementary Figure 18: UV-Vis spectroscopic analysis of changes of the IVCT absorption band (detected at λ = 955 nm) when the Mg^{2+} / {V_{12}} reaction mixture or the {V_{12}} reference is irradiated with monochromatic LEDs (405 nm or 470 nm). Conditions: solvent: acetonitrile, [{V_{12}}] = 0.05 mM, [Mg] = 0.21 mM.

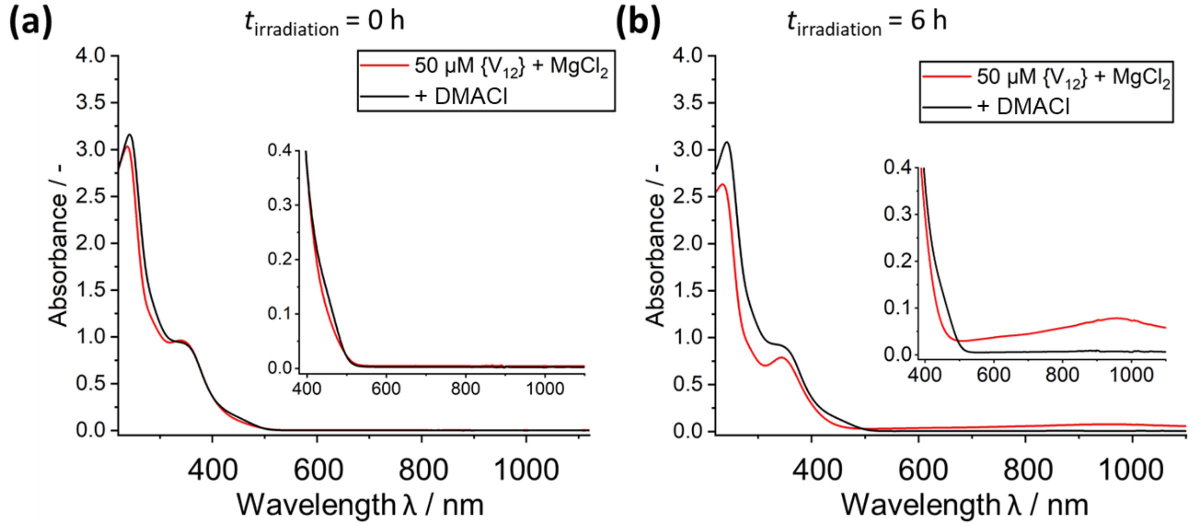

Supplementary Figure 19: UV-Vis-NIR spectra of the {V_{12}} + $MgCl_2$ reaction mixture in the presence and absence of DMACl. (a) before irradiation; (b) after 6 h irradiation with a broadband high-power LED light source ($P_{nominal}$ = 20 W). In the presence of DMACl, no reduced vanadate species are formed (indicated by the absence of the characteristic IVCT band). We propose that this indicates the presence of an excess of DMA cations affects the {V_{12}} / {MgV_{12}} equilibrium, prevents the formation of the photoactive {MgV_{12}} and can therefore be used as a supramolecular control parameter to trigger or prevent vanadate photoreduction. Conditions: solvent: acetonitrile, [{V_{12}}] = 50 µM, [$MgCl_2$] = 211 µM,Ar atmosphere.

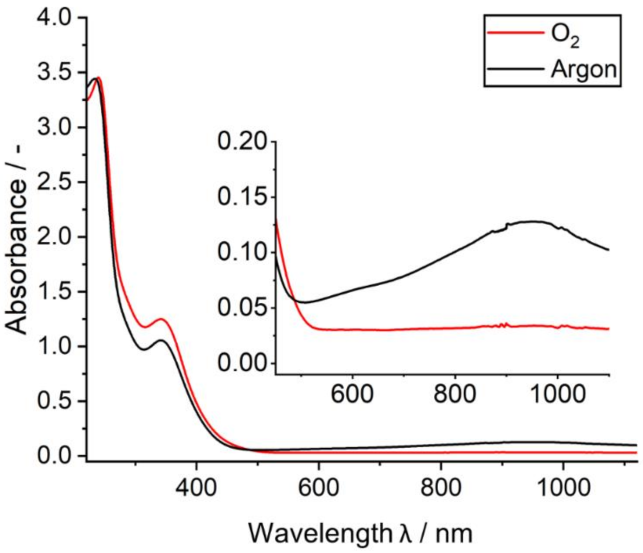

Supplementary Figure 20: UV/Vis spectrum comparison of {V_{12}} + $MgCl_2$ when irradiated with a broadband high-power LED light source ($P_{nominal}$ = 20 W) after 6 hours. Conditions: solvent: acetonitrile, [{V_{12}}] = 50 µM, [$MgCl_2$] = 211 µM.

3.3. Electrochemistry

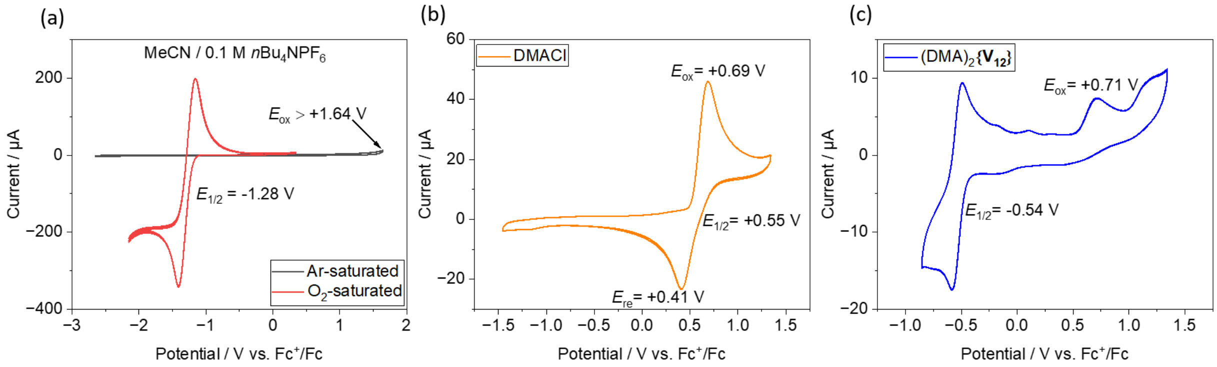

Supplementary Figure 21: Cyclic voltammetry analysis. (a): CV analysis of the electrolyte when purged with Ar or O_2. (b) CV analysis of DMACl (Ar atmosphere). (c) CV analysis of $(DMA)_2${V_{12}} (Ar atmosphere). Conditions: solvent: anhydrous MeCN containing 0.1 M $n$$Bu_4NPF_6$; [DMACl] = 2 mM, [$(DMA)_2${$V_{12}$}] = 1 mM; samples referenced against Fc^+/Fc as internal standard. Scan rate: 100 mV/s.

3.4. Mg^{2+} replacement with Ca^{2+}

To understand whether the observed reactivity between Mg^{2+} and {V_{12}} is unique for Mg^{2+} or can be transferred to other metal cations, identical experiments for metal-functionalization of {V_{12}} were performed using $CaCl_2$ x 2 H_2O instead of $MgCl_2$. The samples were then irradiated, and the reactions were followed by ^{51}V NMR and UV-Vis-NIR spectroscopy. In sum, ^{51}V NMR spectrum show the characteristic four-line signal pattern indicating formation of {CaV_{12}} upon Ca^{2+} addition to {V_{12}}. Irradiation of the reaction solution containing this species with visible light results in the formation of the IVCT band (between ~600-1100 nm) as observed also for the formation of {Mg_2V_{12}}, see details in main manuscript. The experimental data are shown below.

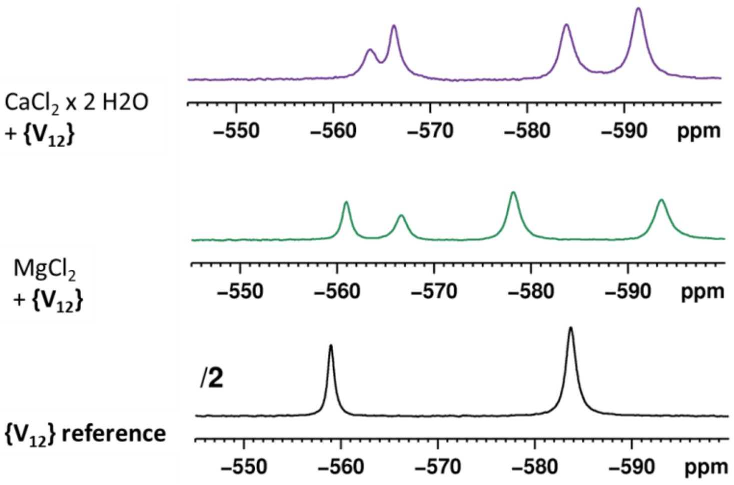

Supplementary Figure 22: ^{51}V NMR spectra of {V_{12}} upon addition of $MgCl_2$ and upon addition of $CaCl_2$ x 2 H_2O under otherwise identical reaction conditions. In both cases, the characteristic four-line signal indicating the formation of the mono-metal-functionalized species (i.e., {MgV_{12}} or {CaV_{12}}) is observed. Conditions: [Mg^{2+}] ca. 21 mM, [Ca^{2+}] ca. 21 mM, [{V_{12}}] ca. 5.0 mM, solvent: CH_3CN.

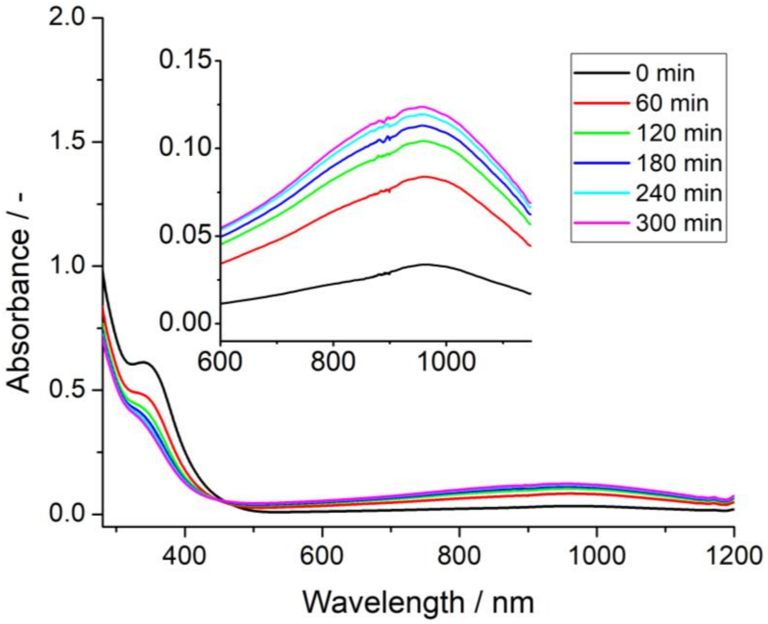

Supplementary Figure 23: UV-Vis-NIR spectra showing the formation of the characteristic IVCT band indicative of formation of a reduced vanadate cluster upon irradiation of a DMF solution containing Ca^{2+} and {V_{12}}. Conditions: [Ca^{2+}] ca. 0.21 mM, [{V_{12}}] ca. 0.05 mM, solvent: DMF.

4. Theoretical calculations

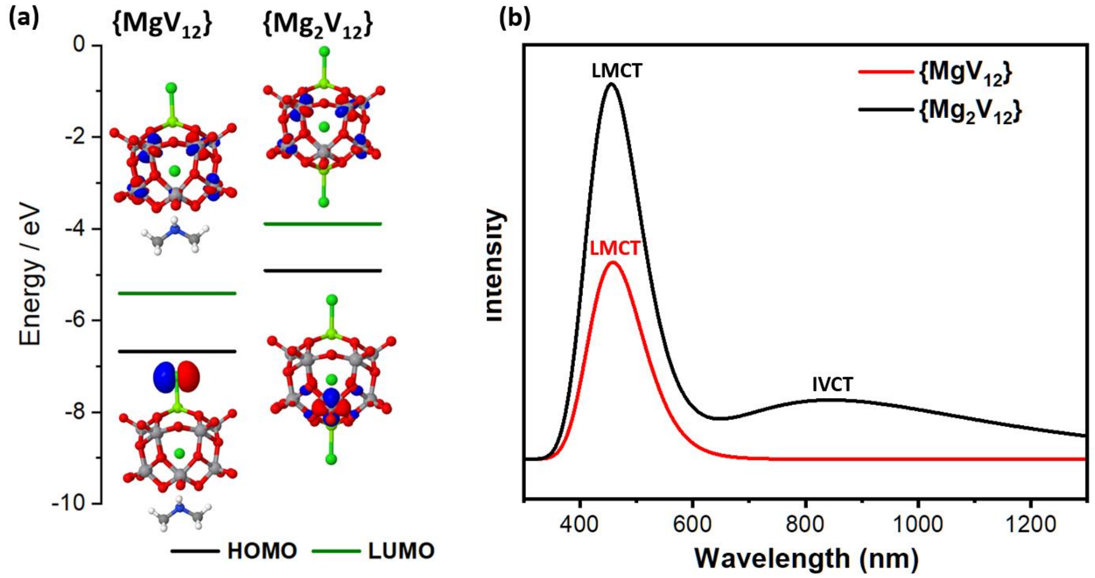

Supplementary Figure 24: Left: Calculated HOMO-LUMO levels of {MgV_{12}} and {Mg_2V_{12}}. **Right:** Calculated UV-Vis-NIR spectra for {MgV_{12}} and {Mg_2V_{12}}.

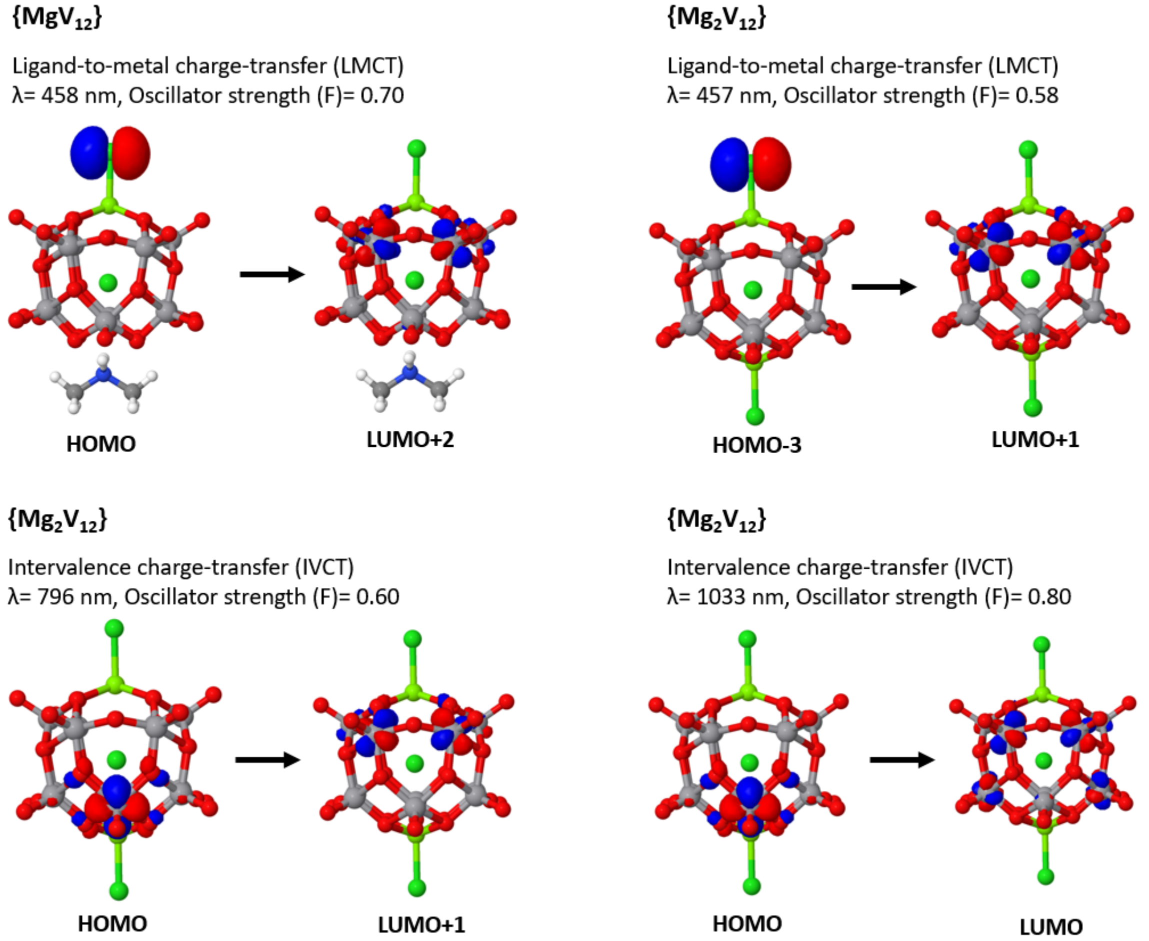

Supplementary Figure 25: Top: orbital illustrations for the calculated high oscillator-strength LMCT transitions in {MgV_{12}} and {Mg_2V_{12}}. **Bottom:** orbital illustrations for the calculated high oscillator-strength IVCT transitions in {Mg_2V_{12}}.

5. References

1. Momma, K. & Izumi, F. VESTA 3 for three-dimensional visualization of crystal, volumetric and morphology data. *J. Appl. Crystallogr.* **44**, 1272–1276 (2011).

2. Brown, I. D. & Altermatt, D. Bond-valence parameters obtained from a systematic analysis of the Inorganic Crystal Structure Database. *Acta Crystallogr. B* **41**, 244–247 (1985).

3. Becke, A. D. Density-Functional Thermochemistry. III. The Role of Exact Exchange. *J. Chem. Phys.* **98**, 5648–5652 (1993).

4. Lee, C., Yang, W. & Parr, R. G. Development of the Colle-Salvetti correlation-energy formula into a functional of the electron density. *Phys. Rev. B* **37**, 785–789 (1988).

5. Weigend, F. & Ahlrichs, R. Balanced basis sets of split valence, triple zeta valence and quadruple zeta valence quality for H to Rn: Design and assessment of accuracy. *Phys. Chem. Chem. Phys.* **7**, 3297 (2005).

6. Marenich, A. V., Cramer, C. J. & Truhlar, D. G. Universal solvation model based on solute electron density and on a continuum model of the solvent defined by the bulk dielectric constant and atomic surface tensions. *J. Phys. Chem. B* **113**, 6378–6396 (2009).

7. Barone, V. & Cossi, M. Quantum calculation of molecular energies and energy gradients in solution by a conductor solvent model. *J. Phys. Chem. A* **102**, 1995–2001 (1998).

8. M. J. Frisch, G. W. Trucks, H. B. Schlegel, G. E. S. *et al.* Gaussian 16, Revision A.03. *Gaussian 16, Revision A.03, Gaussian, Inc., Wallingford CT* (2016).

9. Kastner, K., Margraf, J. T., Clark, T. & Streb, C. A molecular placeholder strategy to access a family of transition-metal-functionalized vanadium oxide clusters. *Chem. Eur. J.* **20**, 12269-12273 (2014).

10. Wilson, E. F. *et al.* Probing the self-assembly of inorganic cluster architectures in solution with cryospray mass spectrometry: growth of polyoxomolybdate clusters and polymers mediated by silver (I) ions. *J. Am. Chem. Soc.* **130**, 13876–13884 (2008).

11. Sheldrick, G. M. Crystal structure refinement with SHELXL. *Acta Crystallogr. C Struct. Chem.* **71**, 3–8 (2015).

12. Dolomanov, O. V., Bourhis, L. J., Gildea, R. J., Howard, J. A. K. & Puschmann, H. OLEX2 : a complete structure solution, refinement and analysis program. *J. Appl. Crystallogr.* **42**, 339–341 (2009).

13. Blessing, R. H. An empirical correction for absorption anisotropy. *Acta Crystallogr. A* **51**, 33–38 (1995).
